# Cause for coercion: cause for concern?

**DOI:** 10.1007/s40592-024-00187-1

**Published:** 2024-02-13

**Authors:** Maxwell J. Smith

**Affiliations:** Western Research Chair in Public Health Ethics, Faculty of Health Sciences, Rotman Institute of Philosophy, Western University, London, ON Canada

**Keywords:** Coercion, Public health, Necessity, Public health ethics

## Abstract

In his 2000 book, *From Chaos to Coercion: Detention and the Control of Tuberculosis*, Richard Coker makes a number of important observations and arguments regarding the use of coercive public health measures in response to infectious disease threats. In particular, Coker argues that we have a tendency to neglect public health threats and then demand immediate action, which can leave policymakers with fewer effective options and may require (or may be perceived as requiring) more aggressive, coercive measures to achieve public health goals. While Coker makes a convincing case as to why we should find it ethically problematic when governments find themselves in this position and resort to coercion, left outstanding is the question of whether this should *preclude* governments and health authorities from using coercion if and when they do find themselves in this position. In this paper, I argue that, while we should consider it ethically objectionable when governments resort to coercion because they have neglected a public health threat, its causes, and other possible responses to that threat, this should not then necessarily *rule out* the use of coercion in such circumstances; that there are ethically objectionable antecedents for why coercion is being considered should not *necessarily* or *automatically* cause us to think coercion in such cases cannot be justified. I address an objection to this argument and draw several conclusions about how governments’ use of coercion in public health should be evaluated.

In his 2000 book, *From Chaos to Coercion: Detention and the Control of Tuberculosis*, Richard Coker examines the causes and responses to the tuberculosis epidemic that plagued New York City in the late 1980s and early 1990s (Coker 2000). By evaluating health authorities’ use of detention and directly observed therapy among so-called ‘recalcitrant’ individuals, Coker makes a number of important observations and arguments regarding the use of coercive public health measures in response to infectious disease threats. In this paper, I shall engage with just three of Coker’s observations in service of further probing the justification for coercion in public health.

First, Coker suggests that only by examining the causes of an epidemic, and our response to it, can we hope to draw lessons, e.g., about the justified use of coercive measures. Hence, the *cause* for coercion should play an important role in our analysis of its justification. The use of coercion ought not to be justified nor condemned in the abstract.

Second, a key theme of Coker’s analysis, as articulated so succinctly in Tim Westmoreland’s foreword to the book, is that coercive measures may be *easier*. That is, single-purpose, coercive interventions may seem less complex to policymakers when compared with interventions that aim to address complicated determinants of health and health behaviours, understand people’s relationship with the health system, facilitate people’s ability to behave like ‘model citizens’, provide for people’s comprehensive care (especially those most in need), and so forth.^1^ Policymakers may favour such measures for this reason.

Finally, we have a tendency to neglect public health threats and then demand immediate action. This can leave policymakers with fewer effective options and may require (or may be perceived as requiring) more aggressive, coercive measures to achieve public health goals. Regarding the tuberculosis epidemic in New York City, Coker remarks:

“The public health system in New York City had failed to detect and act on increasing tuberculosis rates, failed to grasp the causes or understand the implications, and failed to provide for those most in need until 1992. The advice of experts in previous years had been disregarded in a futile effort to save money. Patients had fallen between what were sizeable cracks in the system. Furthermore, the fractured medical system, in its bureaucratic tangle, had failed to provide any continuity of care, focusing rather on in-hospital care, particularly for the insured. Policymakers disregarded the threat of communicable diseases to those unable to access long-term care. The social transformation the city underwent during this period compounded the inadequacy of healthcare provision for the poorest. Overcrowding, homelessness, and HIV all combined to provide the perfect milieu for tuberculosis development and transmission…Thus tuberculosis, an eminently treatable disease requiring a “low-tech” response, spread to infect many of the most vulnerable, in settings where institutions should have been obliged to offer protection, with devastating consequences.” (p. 80–81).

In short, the tuberculosis epidemic had been “encouraged by political, public health, and medical neglect” (p. 117), leading to the perceived necessity of coercive public health measures like detention and directly observed therapy. Hence, *from chaos to coercion*.

Coker’s lucid analysis provides an apt diagnosis of some of the reasons why authorities might turn to coercion in public health. And because it is more favourable to avoid coercion if it is possible to do so, Coker’s analysis also provides good reasons as to why governments and health authorities should avoid acting in this way; that is, they should not settle on whatever approach seems ‘easier’ nor neglect public health threats until their control may be achieved only via more coercive measures. They should seek instead to avoid coercion by proactively addressing public health threats, attending to the causes of the conditions from which illness arises, reforming structures that lead to ‘noncompliance’, intervening in the social conditions that may encourage or facilitate behaviours that are conducive to public health, and so forth.

However, as responses to the COVID-19 pandemic have so clearly shown, we are likely to continue to find ourselves in situations where governments have failed in this regard. While Coker makes a convincing case as to why we should find it ethically problematic when governments find themselves in this position and resort to coercion, left outstanding is the question of whether this should *preclude* governments and health authorities from using coercion if and when they do find themselves in this position. It is this narrow question that I shall address in this brief paper.

Before proceeding, I will define what I mean by ‘coercion’ since it is such a rich and contested concept. For the purposes of this paper, I define coercion as a threat taking the form ‘do *x*, or else *y*’, where the choice forced upon the coercee is such that the coercee has no reasonable choice but to do *x*, and where *y* makes the coercee worse off than they ought to be (Wertheimer 1987). With that being said, I do not think one must subscribe to this exact definition of coercion for the analysis presented in this paper to hold or for it to provide insights regarding when coercion may or may not be justified.

## Should coercion be ‘ruled out’?

Given how apt Coker’s diagnosis is regarding some of the problematic reasons why authorities might resort to using coercive measures in public health, it is attractive to infer that governments are not justified in using coercion in such situations. After all, it was their neglect of the public health threat and their disinterest in addressing structural factors and facilitating voluntary behaviours that led to the threat spiraling out of control and for coercive measures to even be considered. But should we be so hasty in arriving at this conclusion? I argue that while we should consider it ethically objectionable when governments resort to coercion as a result of neglecting a public health threat, its causes, and other possible responses to that threat, this should not then necessarily *rule out* the use of coercion if and when we find ourselves in such less-than-ideal circumstances. To be clear, I do not mean to say that coercion *is* justified in such circumstances, but only that the ethically objectionable antecedents for why coercion is now being considered should not *necessarily* or *automatically* cause us to think coercion in such cases is unjustified.

A compelling reason why we should think this is the case is because ruling out coercion in such circumstances could further harm the public, through no fault of their own. The public should not be obliged to live with the consequences, whatever they may be, of failures by governments to act (or act effectively or appropriately), especially if effective means remain at their disposal to protect the public from harm. That we find ourselves in such a situation should be condemned, no doubt. But it does not follow that insult must be added to injury.

Consider the prospect of introducing visitor restrictions in a hospital to curb the spread of a respiratory virus during an epidemic. Hospitals, governments, and public health authorities should work to avoid this option, if possible, given the unintended negative consequences that would likely result from it. Adequate disease control could perhaps be achieved instead via entrance screening, vaccination programs, air filtration and ventilation, policies to stay home when sick, masking, and so forth, which would permit hospital visitation to continue. Moreover, hospitals could be designed such that visitors do not pose a significant risk of infecting others. In other words, a balance could be struck between protecting patients from communicable diseases and encouraging the benefits afforded by hospital visitation. But suppose hospitals, governments, and public health authorities fail to do any of those things, and the introduction of such measures at such a late stage of an epidemic would be neither timely nor effective at achieving public health goals (given the resources required to do so, the time it would take to implement such measures, etc.). Decision-makers might claim that measures like visitor restrictions are the *only* option at that point in time capable of achieving the public health goals sought, or at least that the costs of coercion cannot be avoided in achieving those goals. This would be frustrating to hear, and we would have reason to hold such decision-makers responsible for allowing such a state of affairs to come about. But automatically concluding that visitor restrictions *cannot* then be implemented because of those decision-makers’ previous failures would be a mistake, because doing so would mean giving no weight to the harms that would likely be experienced by the public (and especially those most at risk) as a result of being forced to use less timely and less effective methods of disease control. Authorities made their bed, but it is not so clear that the public should have to lie in it.

Indeed, it could be the case that the use of coercion is *necessary* to effectively address a threat (that is, it may be the *only possible way* to address a threat and achieve the public health goal that is sought, or it may be that the costs of coercion cannot be avoided in achieving the goal that is sought) (Allen and Selgelid 2017). Consequently, at that particular point in time, there may be no equally effective alternative that is less infringing of moral considerations like autonomy, liberty, privacy, justice, and so forth. It would be a shame if such tools were removed from public health’s armamentarium, as it were, because of government’s prior failures.

In fact, governments may have *stronger* obligations to protect the public from harm, even if that involves coercive measures, if it is in fact the government’s actions that were responsible for increased risk of harm to the public. Governments fail in the first instance to protect the public from harm when they fail to address the causes of the conditions from which illness arises, provide for those most in need, reform structures that lead to ‘noncompliance’, intervene in the social conditions that may encourage or facilitate behaviours that are conducive to public health, and so forth. It does not follow that this failure to protect the public from harm should preclude them from protecting the public from harm at a later point in time. It may mean just the opposite. Moreover, because public health is a shared responsibility among many actors, a public health authority that is considering coercive measures may not be responsible for prior neglect of a threat, failures to attend to the causes of the conditions from which illness arises, and so forth. In such cases, it is hard to see why a national public health authority, for example, should be precluded from using coercive measures because regional public health authorities failed to intervene effectively to avoid such a state of affairs. Dereliction of duties among one or more actors should not necessarily restrict the options available to other actors to effectively address public health threats.

## Can coercion be considered'necessary' in such circumstances?

Now, an objection to this argument regards the extent to which the use of coercion should truly be considered ‘necessary’ in such circumstances. If coercion is unnecessary, then there are good reasons not to use it (indeed, necessity is a prominent justificatory condition for public health intervention, including the use of coercion) (Childress et al. 2002; Allen and Selgelid 2017). By not intervening proactively, addressing the causes of the conditions from which illness arises, and so forth, governments may feel they are licensed to use coercion, as coercion will be perhaps the only option available to them at that point in time that is capable of achieving their public health aims. But according to this objection, the government is violating the requirement of necessity because necessity should be considered historically. One should not determine whether a government meets the requirement of necessity merely by looking at the means available to them at the time of dealing with a threat. Rather, one should look at the earlier decisions that made it so that the options available are only those and not others (Schwartz 2020).

Daniel Schwartz (2020) raises this objection when considering necessity in the context of self-defense.^2^ According to Schwartz, the principle of necessity requires that instances of self-defense involve the ‘least harmful defensively effective means’ of thwarting a wrongful threat. If the self-defender has *no choice* but to kill their attacker, then this would count as the least harmful defensively effective means of thwarting the threat to them (indeed, it is the *only* means of thwarting the threat to them). However, if the self-defender intentionally deprives themselves of certain defensive options (e.g., destroying their escape route), such that their only remaining option is to kill their attacker, then we have reason to believe they have violated the requirement of necessity. Schwartz argues that if one forgoes the defensive option recommended by necessity at the first decision node, one will come to be in breach of necessity no matter what choice one makes at the second node. By analogy, if public health authorities forgo interventions recommended by necessity when initially confronted with a public health threat (or, indeed, even in anticipation of a threat), then we should be skeptical of claims that their later use of coercion is necessary. And if their use of coercion is not considered necessary, then we should think it is not justified.

This objection seems to have some force. However, Schwartz provides a helpful response. For the self-defender who has intentionally deprived themselves of defensive options, it would be too hasty to conclude that the moral thing to do would be for the self-defender to passively endure their death—that killing their attacker would *necessarily* be impermissible. This is not an implication of the view: “At the time of the attack she has no other option, and so even if she is in breach of necessity, given that her life is at risk, we cannot blame Defender for shooting her attacker” (p. 597). By analogy, we might also consider the fact that the public is at risk (from a public health threat) to conclude that governments should not be blamed for intervening with the only tools that may at that point in time be capable of addressing the threat (though they might still be blamed for causing that state of affairs in the first place). In other words, governments may be in breach of necessity but should not *necessarily* be blamed for deploying coercive tools, especially when there are no other similarly effective options and when the public is at serious risk of harm.^3^ Governments have an obligation to protect the public’s health, and this obligation does not disappear because they have failed to do so adequately or appropriately at a prior point in time. But this does not mean coercion will necessarily be justified, either. For instance, it is still possible to think it is excessive for governments to use coercion when other equally or more effective measures are available that do not involve coercion. So, coercion should not be ruled out, but that does not mean its use would *necessarily* be justified. Hence, we should assess the permissibility of using coercion in this case much like we might in others.

## Conclusions and implications

This brief argument has a number of implications for how we might think about the use of coercion in public health. First, while governments should not be precluded from using coercion because of their previous failures to adequately or appropriately address a public health threat, they can and should still be held accountable for those failures, including the fact that those failures ultimately ‘necessitated’ the later use of coercion. Adequately and appropriately addressing a public health threat without resorting to coercion represents a more favourable balance between the values of protecting public health and individual liberty. Authorities should therefore be judged and held accountable for a prior lack of appropriate action and their subsequent use of coercive measures, including the less favourable balancing of the values of protecting public health and individual liberty, even if we think *at that point in time* those measures were justified in being used. This is particularly salient as the world attempts to learn lessons from the COVID-19 pandemic. It may be that instances of coercion were ultimately permissible, but governments should still be held accountable for the impacts (e.g., on liberty) that they could have (ostensibly) otherwise avoided. In such cases, the concern is not simply that coercion was used, but that authorities had a reasonable opportunity to avoid a state of affairs where it needed to be.

Second, this argument highlights a potential deficit of ‘least infringement’ or ‘least restrictive alternative’ principles in public health ethics; namely, that they tend to largely ignore considerations of temporality and history. The principle of ‘least infringement’ is commonly taken to mean that public health authorities should seek to minimize the infringement of moral considerations like liberty, autonomy, privacy, justice, and so forth (Allen and Selgelid 2017). The ‘least restrictive alternative’ principle is commonly taken to mean that, among equally effective options, public health authorities should seek the least restrictive alternative (Allen and Selgelid 2017). By failing to intervene proactively, adequately, and appropriately, public health authorities may put themselves in a position where they are now considering the use of coercive tools. And they may ‘apply’ these principles at that point in time and conclude that the coercive tools they are considering are the least infringing or least restrictive alternative among the options available to them. But this is only because they have failed to intervene proactively, adequately, and appropriately to that point. In other words, such principles could be used to license coercive options despite the fact that those options could have been avoided. Consequently, if one thinks the justificatory conditions for the use of coercion should account for the causes that have led authorities to consider it, then such principles may need to be amended or supplemented.

By contrast, Upshur’s (2002) interpretation of the principle of ‘least restrictive means’ has a more explicit temporal dimension insofar as it states that more coercive methods should be employed “only when less coercive methods have failed” (p. 102). But in the sorts of situations with which this paper has been concerned, it is not so much that less coercive methods have failed, but rather that they simply have not been tried at all. To invoke Coker’s (2000) language again, there has been “political, public health, and medical neglect” (p. 117). In such cases, to expect less coercive measures to be tried in the midst of a crisis and proceed only when they have failed seems unduly stringent, and, again, may create an unnecessarily high risk of harm to the public. There is a point at which more coercive methods may be the most prudent and ethically justifiable course of action even when less coercive methods have not been tried, e.g., when we have good reason to believe less coercive methods would fail, when a threat of severe outcomes exists in the absence of coercive countermeasures, when waiting to implement more coercive methods until less coercive options have been found to be ineffective could reasonably be expected to result in significant harms that might otherwise have been avoided, when forgoing coercive measures could be expected to create or exacerbate inequities, etc.

Finally, the argument I have outlined suggests that if one is committed to avoiding coercion in public health, one ought to be committed to the sorts of interventions that could be deployed proactively to avoid its use. Consider, again, the case example of hospital visitor restrictions. If one thinks such policies are objectionable because they involve coercion (or because they are otherwise unduly ‘restrictive’), it would be unhelpful, if not incoherent, to also object to the less coercive policies that could be implemented to avoid more coercive options (e.g., entrance screening, policies that encourage or incentivize vaccination and masking, air filtration and ventilation, policies that ask visitors to stay at home when sick, etc.). In other words, the most stalwart opponents of using coercion in public health should be the strongest proponents of proactive public health intervention. Objecting to coercion in public health without supporting proactive measures that could obviate its use suggests an interest not in questioning the appropriate means of achieving public health goals but in questioning the pursuit of public health goals altogether.

The COVID-19 pandemic was a constant reminder of Coker’s insightful analysis regarding the use of coercion in public health. We must push for governments and health authorities to proactively intervene to detect and act on communicable disease threats, grasp their causes and understand their implications, and provide for those most in need. This will help to avoid resorting to coercive measures to achieve public health aims. Unfortunately, just like during the COVID-19 pandemic, we are likely to continue to find ourselves in situations where authorities have failed in this regard. I have argued that when we do find ourselves in such situations, authorities can and should be held to account, but the ethically objectionable antecedents that may explain why coercion is being considered should not necessarily or automatically cause us to think coercion in such cases is unjustified.

## Footnotes

1 Whether the use of coercive measures is actually ‘easier’ for policymakers, particularly given public sentiments regarding the use of such measures, is a slightly different empirical claim and is likely to be context dependent.

2 My thanks to Nicholas G. Evans for prompting me to consider this line of argument.

3 Another possible response to this objection, also adapted from Schwartz, is to say that the evolution of a threat, like an epidemic, represents multiple temporally separated threats. When an epidemic spirals out of control, for instance, governments may wish to argue they are facing a *new* threat (as opposed to the same threat they faced earlier, perhaps due to a new variant of the pathogen), and that we should therefore only look at the options available in *that* situation for the purposes of the necessity assessment. In other words, governments may wish to claim they did not forfeit certain options when they failed to intervene or when they neglected the threat at an earlier phase of the epidemic, as they expected they would be able to avail themselves of those options at a later point, only to learn they could not because the threat at that later point was different. However, for this response to be forceful, one must be persuaded that some later stage of an epidemic (when coercion is being considered) truly represents a new threat. As Schwartz notes, “[w]e do not think that the passage of time itself makes one threat die and another begin” (pg. 596). A final possible response to this objection offered by Schwartz is to invoke a lesser-evil justification, whereby it can sometimes be permissible (and perhaps even obligatory) to inflict some amount of excessive harm if necessary to avert an even worse harm, despite being in breach of necessity. By analogy, we might think that, despite being in breach of necessity, governments are justified in using coercion (and imposing its attendant harms) if is it necessary to avert an even worse harm.
